# Surveillance of Symptom Burden Using the Patient-Reported Outcome Version of the Common Terminology Criteria for Adverse Events in Patients With Various Types of Cancers During Chemoradiation Therapy: Real-World Study

**DOI:** 10.2196/44105

**Published:** 2023-03-08

**Authors:** Danbee Kang, Sooyeon Kim, Hyunsoo Kim, Mangyeong Lee, Sun-Young Kong, Yoon Jung Chang, Sung Hoon Sim, Yeon-Joo Kim, Juhee Cho

**Affiliations:** 1 Department of Clinical Research Design and Evaluation Samsung Advanced Institute for Health Sciences & Technology Sungkyunkwan University Seoul Republic of Korea; 2 Center for Clinical Epidemiology Samsung Medical Center Sungkyunkwan University School of Medicine Seoul Republic of Korea; 3 Targeted Therapy Branch and Department of Laboratory Medicine National Cancer Center Goyang Republic of Korea; 4 Department of Laboratory Medicine National Cancer Center Goyang Republic of Korea; 5 Cancer Biomedical Science Graduate School of Cancer Science and Policy National Cancer Center Goyang Republic of Korea; 6 Division of Cancer Control & Policy National Cancer Control Institute National Cancer Center Goyang Republic of Korea; 7 Center for Breast Cancer National Cancer Center Goyang Republic of Korea; 8 Department of Radiation Oncology National Cancer Center Goyang Republic of Korea

**Keywords:** surveillance, patient-reported outcome, symptoms, cancer

## Abstract

**Background:**

Over 90% of patients with cancer experience 1 or more symptoms caused directly by cancer or its treatment. These symptoms negatively impact on the completion of planned treatment as well as patients’ health-related quality of life (HRQoL). It often results in serious complications and even life-threatening outcomes. Thus, it has been recommended that surveillance of symptom burden should be performed and managed during cancer treatment. However, differences in symptom profiles in various patients with cancer have not been fully elucidated for use in performing surveillance in the real world.

**Objective:**

This study aims to evaluate the burden of symptoms in patients with various types of cancers during chemotherapy or radiation therapy using the PRO-CTCAE (Patient-Reported Outcome Version of the Common Terminology Criteria for Adverse Events) and its impact on quality of life.

**Methods:**

We performed a cross-sectional study of patients undergoing outpatient-based chemotherapy, radiation therapy, or both at the National Cancer Center at Goyang or at the Samsung Medical Center in Seoul, Korea between December 2017 and January 2018. To evaluate cancer-specific symptom burden, we developed 10 subsets for using the PRO-CTCAE-Korean. To measure HRQoL, we used the European Organization for Research and Treatment of Cancer Core Quality of Life Questionnaire Core 30 (EORTC QLQ-C30). Participants answered questions prior to their clinic appointments on tablets. Multivariable linear regression was used to analyze symptoms based on cancer type and to evaluate the association between the PRO-CTCAE items and the EORTC QLQ-C30 summary score.

**Results:**

The mean age (SD) of the patients was 55.0 (11.9) years, and 39.94% (540/1352) were male. Overall, symptoms in the gastrointestinal category were the most dominant in all cancers. Fatigue (1034/1352, 76.48%), decreased appetite (884/1352, 65.38%), and numbness and tingling (778/1352, 57.54%) were the most frequently reported. Patients reported more local symptoms caused by a specific cancer. In terms of nonsite-specific symptoms, patients commonly reported concentration (587/1352, 43.42%), anxiety (647/1352, 47.86%), and general pain (605/1352, 44.75%). More than 50% of patients with colorectal (69/127, 54.3%), gynecologic (63/112, 56.3%), breast (252/411, 61.3%), and lung cancers (121/234, 51.7%) experienced decreased libido, whereas 67/112 (59.8%) patients with gynecologic cancer and lymphoma/myeloma reported pain during sexual intercourse. Patients with breast, gastric, and liver cancers were more likely to have the hand-foot syndrome. Worsening PRO-CTCAE scores were associated with poor HRQoL (eg, fatigue: coefficient –8.15; 95% CI –9.32 to –6.97), difficulty in achieving and maintaining erection (coefficient –8.07; 95% CI –14.52 to –1.61), poor concentration (coefficient –7.54; 95% CI –9.06 to –6.01), and dizziness (coefficient –7.24; 95% CI –8.92 to –5.55).

**Conclusions:**

The frequency and severity of symptoms differed by cancer types. Higher symptom burden was associated with poor HRQoL, which suggests the importance of appropriate surveillance of PRO symptoms during cancer treatment. Considering patients had comprehensive symptoms, it is necessary to include a holistic approach in the symptom monitoring and management strategies based on comprehensive patient-reported outcome measurements.

## Introduction

Over 90% of patients with cancer experience 1 or more symptoms caused directly by cancer or its treatment [1,2]. These symptoms negatively impact on the completion of planned treatment as well as their health-related quality of life (HRQoL). It often results in serious complications and even life-threatening outcomes [3]. Thus, it has been recommended that surveillance of symptom burden should be performed and managed during cancer treatment [4].

However, barriers to symptom monitoring are medical jargon and lack of trust [5]. In addition, there have been only few reliable and valid tools for this purpose [5]. Thus, there have been growing calls for developing and implementing standardized patient-reported outcome measurements for symptom surveillance in patients with cancers for both clinical care and research purposes [6]. A major advancement in this direction is the US National Cancer Institute’s PRO-CTCAE (Patient-Reported Outcome Version of the Common Terminology Criteria for Adverse Events), which comprises 124 items of 15 categories, based on 78 CTCAE toxicities considered appropriate for patient reporting [7,8]. According to a qualitative study [5], although many participants appreciated the personalized approach with symptom monitoring, they do not want to use a symptom checker that asks too many questions or that takes too long to complete. As frequent administration of the complete library of the PRO-CTCAE is impractical and burdensome, the Food and Drug Administration recommends selecting a relevant item set that can provide insights into the most important toxicities for the treatments being evaluated [9].

So far, most guidelines developed for selecting symptom measures were designed for clinical trials, and there is a lack of guidance for practitioners and for performing surveillance in the real-world clinical setting [10]. In fact, when the adverse events (AEs) were collected from the real world, new side effects related to the medication use not listed on the drug label have been reported [11]. Although a subset for practice was recently suggested based on a Delphi study with stakeholder panels [12,13], they only covered common cancers in Western countries, such as breast, lung, and colorectal cancers; further, the guidelines are limited to uncommon cancers, such as lymphoma, stomach, or liver cancers. In addition, differences in symptom profiles among various patients with cancer have not been fully elucidated for use in performing surveillance in the real world [14]. Therefore, we aimed to evaluate the burden of symptoms in patients with various types of cancers during chemotherapy or radiation therapy using the PRO-CTCAE in the real world and its impact on quality of life.

## Methods

### Study Population

We performed a cross-sectional study of patients undergoing outpatient-based chemotherapy, radiation therapy, or both at the National Cancer Center (NCC) at Goyang or at the Samsung Medical Center (SMC) in Seoul, Korea between December 2017 and January 2018. Eligible participants were (1) older than age 18; (2) diagnosed with cancer; (3) currently receiving chemotherapy or radiation therapy or both; and (4) those who can read, speak, and comprehend Korean. To include a more diverse sample of patients with cancer who have relatively little information about symptom burden, we aimed to recruit at least 50 patients with lymphoma, gastric, gynecologic, head and neck, and liver cancers. To simultaneously evaluate the measurement properties of all items of the PRO-CTCAE-Korean (n=124) within a single study, we aimed to recruit 1300 patients with cancer. Based on the site investigator’s assessment, patients with clinically significant cognitive impairment were excluded from the study. The sampling frame was monitored to ensure that a minimum of 15% of participants had an impaired performance status (PS), defined as an Eastern Cooperative Oncology Group (ECOG) PS of 2 or higher [7].

Participants answered questions prior to their clinic appointments on tablets without assistance but could request technical assistance from the study staff if required.

### Measures

For cancer-specific symptom burden surveillance, we developed 10 subsets of the PRO-CTCAE-Korean (9 for specific cancers and 1 for general purpose). The PRO-CTCAE item library has been previously translated and validated in Korean [15,16]. The PRO-CTCAE-Korean instrument has been linguistically validated for use in Korean-speaking populations [15]. In addition, the instrument has shown high construct validity (correlation [*r*] for all items >0.30 with the anchor items) and high test-retest reliability (range of intraclass correlation coefficient 0.33-0.83) [16]. To generate a subset for each type of cancer, we included common symptomatic AEs recommended by the National Cancer Institute based on their high prevalence across different cancer treatment types [17]. In addition, we included symptomatic AEs that were prevalent in specific subgroups for different cancer sites based on a literature review and recommendation by a panel of 15 medical and radiation oncologists. We performed a Delphi survey 2 times by providing the panel with the entire list of the PRO-CTCAE-Korean instrument and then asking them to choose prevalent and important symptoms to generate subsets for each cancer type. The subsets contained a minimum of 28 to a maximum of 58 symptomatic AEs.

To measure HRQoL, we used the European Organization for Research and Treatment of Cancer Core Quality of Life Questionnaire Core 30 (EORTC QLQ-C30), previously validated in Korean [18], and scored the QLQ-C30 summary score according to the scoring manual [19]. Higher scores on these indicate better function. Further, demographic information was gathered by self-reporting, and clinical variables were obtained from electronic health records.

### Statistical Analysis

A composite-grade scoring algorithm was used to obtain single numerical grades for AEs based on multiple PRO-CTCAE items [20]. Following the composite grading algorithm, a single composite grade was given to the PRO-CTCAE item combinations within the range of 0-3. A higher composite grade indicates the worse symptom experience. One rule of thumb for interpreting the difference in PRO scores is 10% of the instrument range [21]. As the score ranged between 0 and 3, we considered that differences of 0.4 points (ie, 10% of the score range) were clinically meaningful.

The symptom prevalence by composite grades of the PRO-CTCAE items is shown using a tree map. The size of the rectangles in the tree map indicates the proportion of patients with symptoms, with the darker colors indicating a higher prevalence of patients who reported symptoms as severe. Linear regression was used to analyze symptoms based on cancer type. Covariates adjusted were patient’s age, sex, ECOG, and treatment types. Linear regression was also performed to evaluate the association between the PRO-CTCAE items and the EORTC QLQ-C30 summary score [22].

All analyses were performed using STATA version 16 (StataCorp LP) and R 3.6.1 (R Foundation for Statistical Computing). *P* values <.05 were considered statistically significant.

### Ethical Considerations

Study participants provided written informed consent. We gave the participants a US $5 gift card to thank them for their participation. The Institutional Review Board of the Samsung Medical Center (SMC 2020-04-157) and the National Cancer Center (NCC2017-0249) approved this study. All the research data were encrypted.

## Results

A total of 1352 patients (breast, n=411; colorectal, n=127; gastric, n=123; gynecologic, n=112; head and neck, n=56; liver, n=67; lung, n=234; lymphoma, n=112; prostate, n=57; and others, n=53) participated in this study. The mean age (SD) of the patients was 55.0 (11.9) years; 39.94% (540/1352) were male and 79.29% (1072/1352) received chemotherapy (Table 1).

**Figure 1:**
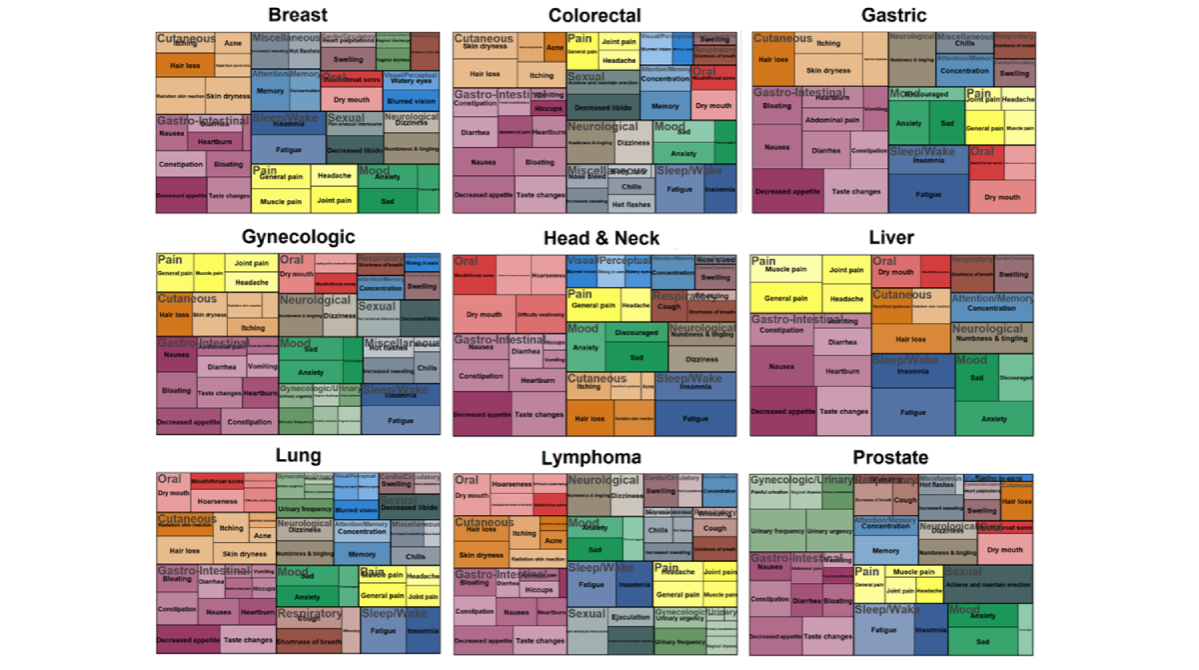
Tree map representing symptom severity and prevalence by type of cancer. Colors indicate the symptom summary score. Sizes of squares are proportion. Thus, the darker the color and greater the size of a square, the more severe and more prevalent the symptom.

Gastrointestinal cancer (purple) was the most dominant among all cancers (Figure 1). Patients commonly reported decreased appetite (884/1352, 65.38%), taste change (764/1352, 56.51%), nausea (588/1352, 43.49%), constipation (607/1352, 44.90%), and diarrhea (459/1352, 33.95%; Figure 1 and Multimedia Appendix 1). Fatigue (1034/1352, 76.48%) was the most frequently reported symptom followed by decreased appetite (884/1352, 65.38%), numbness and tingling (778/1352, 57.54%), insomnia (773/1352, 57.17%), taste change (764/1352, 56.51%), and hair loss (691/1352, 51.11%; Multimedia Appendix 1).

When we compared the symptoms across different types of cancer, patients reported more local symptoms caused by a specific cancer (Multimedia Appendix 1). After adjusting for age, sex, ECOG status, and treatment type, patients with gastric cancer were more likely to have taste changes, decreased appetite, vomiting, diarrhea, and abdominal pain compared with those with other types of cancer. Patients with head and neck cancer had more dry mouth and difficulty in swallowing compared with those with other types of cancer (Multimedia Appendix 2).

In terms of nonsite-specific symptoms, patients commonly reported concentration (587/1352, 43.42%), anxiety (647/1352, 47.86%), sadness (638/1352, 47.19%), and general pain (605/1352, 44.75%; Multimedia Appendix 1). After adjusting for confounders, patients with liver cancer experienced relatively more fatigue and anxiety than those with other types of cancer (Multimedia Appendix 2).

More than 50% of patients with colorectal, gynecologic, breast, and lung cancers and lymphoma/myeloma experienced sexual symptoms, such as decreased libido or pain during sexual intercourse (Multimedia Appendix 1). For example, 49.6% (63/127) of patients with colorectal cancer reported problems in achieving and maintaining erection and 51.7% (121/234) of patients with lung cancer reported decreased libido. Patients with breast (265/411, 64.4%), colorectal (63/127, 49.6%), gastric (68/123, 55.3%), gynecologic (65/112, 58.0%), and lung cancers (103/234, 44.0%) and lymphoma (52/112, 46.4%) more frequently reported skin dryness (Multimedia Appendix 1). Patients with breast (coefficient 0.19; 95% CI 0.09 to 0.28), gastric (coefficient 0.17; 95% CI 0.04 to 0.30), and liver (coefficient 0.21; 95% CI 0.04 to 0.38) cancers were more likely to have the hand-foot syndrome even after adjusting for potential confounders (Multimedia Appendix 2).

We observed a significant decrease in the mean QLQ-C30 summary scores across worsening PRO-CTCAE scores (Multimedia Appendix 3). In particular, memory, all the symptoms in the mood category, fatigue, difficulty in achieving and maintaining erection, body odor, concentration, and dizziness were associated with decreasing of QLQ-C30 summary scores (Multimedia Appendix 3).

**Table 1:** Characteristics of study participants (N=1352).

Characteristics			Breast (n=411)		Colorectal (n=127)		Gastric (n=123)		Gynecologic (n=112)		Head and neck (n=56)		Liver (n=67)		Lung (n=234)		Lymphoma (n=112)		Prostate (n=57)		Others (n=53)		*P* value
Age group, mean (SD)			49.1 (9.4)		56.4 (9.6)		56 (11.4)		52.8 (11)		58.9 (11.3)		59.7 (9.4)		61 (9.4)		52.9 (16.5)		67.8 (10.2)		55.9 (13.2)		<.01
**Gender, n (%)**																							<.01
	Female	409 (99.5)		59 (46.5)		40 (32.5)		112 (100)		18 (32.1)		23 (34.3)		82 (35.0)		40 (35.7)		8 (14.0)		21 (39.6)			
	Male	2 (0.5)		68 (53.5)		83 (67.5)		0 (0)		38 (67.9)		44 (65.7)		152 (65.0)		72 (64.3)		49 (86.0)		32 (60.4)			
**Education, n (%)**																							<.01
	Less than middle school	36 (8.8)		27 (21.3)		28 (22.8)		22 (19.6)		17 (30.4)		16 (23.8)		79 (33.8)		22 (19.6)		19 (33.3)		9 (17.0)			
	High school	170 (41.4)		51 (40.2)		51 (41.5)		51 (45.5)		16 (28.6)		27 (40.3)		102 (43.6)		46 (41.1)		19 (33.3)		19 (35.8)			
	More than college	205 (49.9)		49 (38.6)		44 (35.8)		39 (34.8)		23 (41.1)		24 (35.8)		53 (22.6)		44 (39.3)		19 (33.3)		25 (47.2)			
**Employment status, n (%)**																							<.01
	Employed	110 (26.8)		40 (31.5)		44 (35.8)		11 (9.8)		15 (26.8)		15 (22.4)		58 (24.8)		37 (33.0)		8 (14.0)		15 (28.3)			
	Unemployed	301 (73.2)		87 (68.5)		79 (64.2)		101 (90.2)		41 (73.2)		52 (77.6)		176 (75.2)		75 (67.0)		49 (86.0)		38 (71.7)			
**Monthly family income, n (%)**																							<.01
	<US $1990	75 (18.2)		28 (22.0)		44 (35.8)		27 (24.1)		14 (25.0)		24 (35.8)		84 (35.9)		29 (25.9)		27 (47.4)		9 (17.0)			
	US $2000-US $3990	153 (37.2)		62 (48.8)		45 (36.6)		55 (49.1)		26 (46.4)		26 (38.8)		97 (41.5)		41 (36.6)		21 (36.8)		26 (49.1)			
	≥US $4000	183 (44.5)		37 (29.1)		34 (27.6)		30 (26.8)		16 (28.6)		17 (25.4)		53 (22.6)		42 (37.5)		9 (15.8)		18 (34.0)			
**ECOG^a^ performance status at the first visit, n (%)**																							.01
	0-1	347 (84.4)		111 (87.4)		87 (70.7)		84 (75.0)		47 (83.9)		57 (85.1)		190 (81.2)		89 (79.5)		50 (87.7)		42 (79.2)			
	2-4	64 (15.6)		16 (12.6)		36 (29.3)		28 (25.0)		9 (16.1)		10 (14.9)		44 (18.8)		23 (20.5)		7 (12.3)		11 (20.8)			
**Current treatment, n (%)**																							<.01
	Chemotherapy	306 (74.5)		105 (82.7)		123 (100)		94 (83.9)		25 (44.6)		60 (89.6)		182 (77.8)		109 (97.3)		32 (56.1)		36 (67.9)			
	Radiation	81 (19.7)		3 (2.4)		0 (0)		6 (5.4)		15 (26.8)		4 (6.0)		13 (5.6)		1 (0.9)		19 (33.3)		15 (28.3)			
	Both	24 (5.8)		19 (15)		0 (0)		12 (10.7)		16 (28.6)		3 (4.5)		39 (16.7)		2 (1.8)		6 (10.5)		2 (3.8)			

^a^ECOG: Eastern Cooperative Oncology Group.

## Discussion

In this large real-world surveillance study of burden of symptoms among patients with various types of cancers undergoing chemoradiation therapy, frequency and severity of symptoms differed by cancer types. A higher symptom burden score was associated with poor quality of life, suggesting the importance of appropriate surveillance of PRO symptoms during cancer treatment.

Symptoms in the gastrointestinal category were most commonly reported among patients with different cancer types than those in other categories, and this finding is consistent with the results of a previous study [23]. Gastrointestinal cancer symptoms reportedly plagued many patients regardless of their cancer types [23] because chemotherapy can damage healthy cells in the lining of the digestive system from the mouth to the anus [24]. Therefore, chemotherapy can affect these areas and cause vomiting, diarrhea, constipation, and mouth sores. The most common side effects of chemotherapy associated with the gastrointestinal tract are taste changes, chemotherapy-induced nausea and vomiting, constipation, and diarrhea [25]. The prevention of chemotherapy-induced nausea and vomiting has been revolutionized over the past few years. Vomiting can be prevented in most cases [25]. However, other factors such as advanced age, decreased mobility, dietary errors, psychological alterations, and cancer-related complications may increase its occurrence [26]. In clinical practice, the implementation of nonpharmacological strategies plays an important role as an adjunct to pharmacological agents in alleviating chemotherapy-induced gastrointestinal symptoms.

In this study, we found that patients experienced more frequent and more severe symptoms caused by a specific cancer. For example, head and neck as well as lung and prostate cancers had relatively more oral, respiratory, and urinary tract symptoms, respectively, compared with other types of cancer. Our study findings are somewhat similar to the results of previous studies which reported that most patients (>80%) experienced symptoms related to their cancer site [27]. Although chemotherapy and related treatment regimens affect the whole body, the origin site of cancer seems to have a greater impact on patient’s symptom burden. As the different types of cancer had different symptoms even when patients received the same treatment, it is necessary to provide specific care to manage the complexity of the symptoms by the cancer type [28]. Therefore, it is important to develop and use a specific subset of the assessment when evaluating chemotherapy- or radiation-induced symptoms for patients with different types of cancers or undergoing treatment options.

Using the PRO-CTCAE will help detect symptoms that were often underreported by health professionals [29], as we have shown in this study, where many patients reported problems with sexual dysfunction, which was not often assessed in previous studies [17]. In our study, more than 50% of patients with colorectal (69/127, 54.3%), gynecologic (63/112, 56.3%), breast (252/411, 61.3%), and lung cancers (121/234, 51.7%) experienced decreased libido, whereas 67/112 (59.8%) patients with gynecologic cancer and lymphoma/myeloma reported pain during sexual intercourse. However, these sexual dysfunctions are often overlooked in evaluating patients with cancers [30]. The causes of sexual dysfunction are psychological distress and endocrine dysfunction caused by the cancer itself or side effects of anticancer treatments such as surgery, radiotherapy, chemotherapy, and hormonal therapy. For example, among patients with colorectal cancer, the rates of sexual dysfunction can be even higher given the physiological changes that can result from surgery, chemotherapy, and radiation therapy [31]. Similarly, surgery for lung cancer may adversely affect the psychogenic status and sexual function in patients with lung cancer due to its invasive nature [32]. As cancer treatments and the emotional suffering from a cancer diagnosis can affect all aspects of sexuality [33], sexuality should be a routine assessment, and communication should begin early on [34].

The PRO-CTCAE includes 6 symptoms of cutaneous toxicity, which were frequently reported by our patients with lymphoma/myeloma, colorectal, gynecologic, gastric, breast, and lung cancers. Although cutaneous toxicities are the common side effects reported by patients with cancer receiving chemotherapy [35], they are often considered minor complaints compared with other side effects such as nausea or vomiting [36]. However, cutaneous toxicity is strongly associated with psychological well-being and HRQoL. Most patients with cancer reported that the impact of chemotherapy on skin irritation and dry skin was worse than they had anticipated [37]. More than two-thirds of patients that developed cutaneous side effects due to chemotherapy were significantly distressed by their altered appearance [38]. It disturbs their daily activities and personal relationships and negatively impacts their HRQoL [39]. Considering the burden of cutaneous symptoms on patients’ daily activities and HRQoL [40], a multidisciplinary cancer care team, including dermatologists, oncologists, and nurses, should perform regular surveillance and appropriate interventions. Furthermore, cutaneous toxicity needs to be monitored more actively in patients with targeted agents, as dermatological toxicities are among the most common complications of targeted agents [41].

Regarding the impact of symptoms burden on HRQoL, most symptoms were associated with lower HRQoL. In particular, memory, mood, fatigue, erection, body odor, concentration, and dizziness were associated with clinically noticeable declines in HRQoL. As these symptoms were associated with daily life, they might have a greater impact on HRQoL due to their burden. In particular, fatigue was reported as one of the most common side effects of cancer that was associated with poor HRQoL, which is similar to the finding reported in a previous study [42]. Fatigue negatively impacts work, social relationships, mood, and daily activities and causes significant impairment in the overall HRQoL during and after treatment [42]. Regarding mood, all patients with cancer had a certain level of anxiety, which could be related to the fear of treatment or fear of cancer recurrence. Considering that anxiety as a symptom is dynamic and can change over time in response to cancer-related events [43], surveillance and treatment planning should incorporate factors contributing to anxiety and patient preference for psychiatric care [43]. Pain is also a frequent, nonsite-specific symptom in all patients. A more active approach may be necessary to manage pain, as it could affect many other symptoms. Patients experienced loss of confidence and restriction in physical and social activities due to chronic dizziness [44]. The burden of shortness of breath might be further compounded by fatigue, anxiety, and depression, resulting in functional limitations and compromised HRQoL [45]. Patients with cancer reported more distress when they experienced a symptom they did not anticipate [37]. Therefore, it is important to provide pretreatment information, counseling, and management resources concerning possible treatment-related side effects in these patients.

There are several limitations to our study. First, the reporting of the symptom scores was voluntary. Thus, the receipt of symptom screening may itself bias the estimates of symptom burden because the routine collection of PROs is associated with improved clinical outcomes and increased patient satisfaction. In addition, patient factors, including male sex and advanced age, were associated with lower rates of PRO-CTCAE reporting, which could reflect differential rates of participation among patient subgroups. Second, as symptom assessments are only recorded at outpatient visits, we did not capture the symptoms of patients who are admitted to the hospital or hospice, or who are otherwise too unwell to visit clinics and may probably be the most symptomatic. In addition, because of the heterogeneity in our cohort, we did not assess the influence of treatment modalities, which will differ substantially among stages, on symptom burden. Finally, although we compared symptom burden based on disease site groups, we did not describe the symptoms of unique cancers, which may mask heterogeneity in the symptom profiles of distinct cancers within larger categories, such as lymphoma/myeloma, colorectal, head and neck/esophageal, prostate/bladder, and gynecologic cancers. Despite these limitations, this study provides guidance on symptoms that should be asked about to patients in the real-world clinical setting. This study also illustrated the feasibility of linking routinely collected PROs to large population-based health care databases.

In conclusion, the frequency and severity of symptoms differed according to the type of cancer, and the symptoms were associated with poor HRQoL. Recently, there has been an emphasis on the appropriate assessment of PRO symptoms during cancer treatment [5,46]. Considering that patients would have various symptoms at different periods, and that symptoms could affect not only disease but also HRQoL, it is necessary to take a holistic approach when implementing a symptom monitoring and management strategy. The results of this study would help physicians to improve their understanding of the variations in cancer treatment–related symptoms and to use proper symptoms list by types of cancer in routine care for developing management plans and guidelines.

## Appendix

Multimedia Appendix 1 Symptom frequency by composite grades of PRO-CTCAE items. PRO-CTCAE: Patient-Reported Outcome Version of the Common Terminology Criteria for Adverse Events.

Multimedia Appendix 2 Difference (95% CI) of cancer-specific symptoms by types of cancer compared with other cancers.

Multimedia Appendix 3 Association between symptoms and health-related quality of life in different types of cancer.

## Abbreviations

AE: adverse event
ECOG: Eastern Cooperative Oncology Group
EORTC QLQ-C30: European Organization for Research and Treatment of Cancer Core Quality of Life Questionnaire Core 30
HRQoL: health-related quality of life
NCC: National Cancer Center
PRO-CTCAE: Patient-Reported Outcome Version of the Common Terminology Criteria for Adverse Events
PS: performance status
SMC: Samsung Medical Center

## Data Sharing

The data sets generated or analyzed during this study are available from the corresponding author (JC) on reasonable request.
