# Supplementary material for: Surveillance of Symptom Burden Using the Patient-Reported Outcome Version of the Common Terminology Criteria for Adverse Events in Patients With Various Types of Cancers During Chemoradiation Therapy: Real-World Study
Source: JMIR Public Health Surveill. 2023 Mar 8;9:e44105. doi: 10.2196/44105 (PMC10034615; doi:10.2196/44105)
Supplement: Multimedia Appendix 1 [file publichealth_v9i1e44105_app1.docx]

| Symptom prevalence | Overall | Breast | Colorectal | Gastric | Gynecologic | Head & Neck | Lymphoma | Liver | Lung | Prostate | Others |
| --- | --- | --- | --- | --- | --- | --- | --- | --- | --- | --- | --- |
| ≥75% | Fatigue  (76.48) | Fatigue  (83.21) |  | Decreased appetite  (79.67) | Fatigue  (85.71) | Fatigue  (75.00) |  | Fatigue  (83.58) |  | Achieve and maintain erection  (75.00) |  |
|  |  |  |  | Fatigue  (78.05) |  |  |  |  |  |  |  |
| 50 ~ <75% | Decreased appetite  (65.38) | Decreased appetite  (70.56) | Fatigue | Taste changes (72.36) | Numbness & tingling | Decreased appetite | Fatigue | Decreased appetite | Fatigue | Fatigue | Fatigue |
|  |  |  | (68.50) |  | (72.32) | (67.86) | (69.64) | (68.66) | (69.23) | (70.18) | (66.04) |
|  | Numbness & tingling  (57.54) | Radiation skin reaction  (70.48) | Decreased appetite | Numbness & tingling | Insomnia (66.07) | Dry mouth (62.50) | Pain w/sexual intercourse (60.00) | Insomnia | Decreased appetite | Urinary frequency (53.70) | Decreased appetite |
|  |  |  | (66.14) | (65.04) |  |  |  | (59.70) | (59.83) |  | (56.60) |
|  | Insomnia (57.17) | Insomnia (66.18) | Numbness & tingling | Insomnia (59.35) | Decreased appetite | Taste changes (62.50) | Numbness & tingling | Anxiety | Shortness of breath |  |  |
|  |  |  | (60.63) |  | (64.29) |  | (58.04) | (56.72) | (53.85) |  |  |
|  | Taste change (56.51) | Numbness & tingling | Taste changes (55.12) | Hair loss (57.72) | Pain w/sexual intercourse (60.00) | Insomnia (58.93) | Decreased appetite | Taste changes (56.72) | Taste changes (52.56) |  |  |
|  |  | (65.94) |  |  |  |  | (53.57) |  |  |  |  |
|  | Hair loss (51.11) | Skin dryness (64.48) | Decreased libido*  (54.55) | Dry mouth (55.28) | Hair loss (59.82) | Anxiety | Insomnia (51.79) | Nausea | Hair loss (52.14) |  |  |
|  |  |  |  |  |  | (50.00) |  | (53.73) |  |  |  |
|  |  | Memory  (61.80) | Hair loss (53.54) | Nausea | Constipation (58.04) | Difficulty swallowing (50.00) |  | Numbness & tingling | Insomnia (52.14) |  |  |
|  |  |  |  | (55.28) |  |  |  | (53.73) |  |  |  |
|  |  | Decreased libido | Achieve and maintain erection* (50.00) | Skin dryness (55.28) | Skin dryness (58.04) |  |  | Concentration (50.75) | Decreased libido* |  |  |
|  |  | (61.22) |  |  |  |  |  |  | (51.61) |  |  |
|  |  | Taste changes (60.34) |  |  | Anxiety |  |  |  |  |  |  |
|  |  |  |  |  | (56.25) |  |  |  |  |  |  |
|  |  | Dry mouth (57.42) |  |  | Sad |  |  |  |  |  |  |
|  |  |  |  |  | (56.25) |  |  |  |  |  |  |
|  |  | Sad |  |  | Decreased libido |  |  |  |  |  |  |
|  |  | (56.20) |  |  | (56.00) |  |  |  |  |  |  |
|  |  | General pain (55.47) |  |  | Dizziness (55.36) |  |  |  |  |  |  |
|  |  | Muscle pain (55.72) |  |  | Bloating |  |  |  |  |  |  |
|  |  |  |  |  | (55.36) |  |  |  |  |  |  |
|  |  | Anxiety |  |  | Dry mouth (54.46) |  |  |  |  |  |  |
|  |  | (54.99) |  |  |  |  |  |  |  |  |  |
|  |  | Hair loss (53.53) |  |  | General pain (54.46) |  |  |  |  |  |  |
|  |  | Pain w/sexual intercourse (52.81) |  |  | Nausea |  |  |  |  |  |  |
|  |  |  |  |  | (53.57) |  |  |  |  |  |  |
|  |  | Increased sweating (52.07) |  |  | Taste changes (52.68) |  |  |  |  |  |  |
|  |  | Constipation (50.12) |  |  |  |  |  |  |  |  |  |
| 25 ~ <50% | Dry mouth (49.93) | Concentration (49.39) | Nausea | Concentration (47.97) | Increased sweating (49.11) | Sad | Dry mouth (48.21) | Hair loss | Numbness & tingling | Decreased appetite | General pain (45.28) |
|  |  |  | (49.61) |  |  | (48.21) |  | (49.25) | (46.15) | (45.61) |  |
|  | Anxiety | Swelling (49.15) | Skin dryness* (49.59) | Bloating | Muscle pain (45.54) | General pain (48.21) | Taste changes (48.21) | General pain (46.27) | Cough | Urinary urgency (44.44) | Anxiety |
|  | (47.86) |  |  | (47.15) |  |  |  |  | (46.12) |  | (45.28) |
|  | Sad | Itching | Dizziness* (47.97) | Diarrhea (45.53) | Concentration (42.86) | Constipation (46.43) | Skin dryness* (46.30) | Sad | Dry mouth (44.87) | Taste changes (43.86) | Constipation (43.40) |
|  | (47.19) | (47.20) |  |  |  |  |  | (43.28) |  |  |  |
|  | Constipation (44.90) | Joint pain (47.93) | Insomnia (45.67) | Anxiety | Heartburn (41.96) | Dizziness* (46.00) | Hair loss (43.75) | Constipation (43.28) | Constipation (44.44) | Sad | Taste changes (43.40) |
|  |  |  |  | (45.53) |  |  |  |  |  | (43.86) |  |
|  | General pain (44.75) | Blurred vision* (45.99) | Memory | Sad | Headache (41.07) | Mouth/  throat sores | Dizziness* (43.52) | Muscle pain* (41.79) | Radiation skin reaction | Memory | Sad |
|  |  |  | (42.28) | (39.84) |  | (44.64) |  |  | (44.23) | (42.59) | (43.40) |
|  | Nausea | Dizziness (45.26) | Bloating | Constipation (35.77) | Diarrhea (40.18) | Hair loss (44.64) | Urinary frequency (42.99) | Heartburn* (37.31) | Skin dryness (44.21) | Anxiety | Radiation skin reaction* (41.18) |
|  | (43.49) |  | (42.28) |  |  |  |  |  |  | (36.84) |  |
|  | Concentration (43.42) | Hot flashes (45.01) | Dry mouth (39.37) | Abdominal pain | Shortness of breath | Numbness & tingling | Achieve and maintain erection | Discouraged (34.33) | Anxiety | Insomnia (38.60) | Dry mouth (39.62) |
|  |  |  |  | (35.77) | (39.29) | (41.07) | (40.74) |  | (43.16) |  |  |
|  | Shortness of breath | Bloating* (45.01) | Diarrhea (39.37) | Itching | Swelling (37.50) | Concentration (39.29) | General pain (40.18) | Dry mouth (34.33) | Dizziness (42.92) | Dry mouth (38.60) | Insomnia (39.62) |
|  | (38.46) |  |  | (37.40) |  |  |  |  |  |  |  |
|  | Diarrhea (33.95) | Nausea | Anxiety | Hand-foot syndrome (34.96) | Joint pain (37.50) | Radiation skin reaction | Sad | Shortness of breath | Memory* (42.92) | Numbness & tingling | Nausea |
|  |  | (44.04) | (38.58) |  |  | (38.71) | (40.18) | (34.33) |  | (33.33) | (37.74) |
|  | Swelling (33.58) | Shortness of breath | Sad | General pain (34.15) | Abdominal pain | Nausea | Constipation (39.29) | Diarrhea | Concentration (41.45) | Constipation (33.33) | Heartburn* (35.85) |
|  |  | (42.58) | (38.58) |  | (36.04) | (37.50) |  | (32.84) |  |  |  |
|  | Mouth/  throat sores | Discouraged (40.88) | Concentration (37.80) | Heartburn (33.33) | Itching | Cracking at the corners of the mouth | Concentration (36.61) | Swelling | Sad | Painful urination (31.48) | Shortness of breath |
|  | (31.66) |  |  |  | (35.71) | (36.00) |  | (31.34) | (41.45) |  | (32.08) |
|  | Headache (30.18) | Watery eyes* (40.88) | Constipation (37.01) | Chills | Urinary frequency (35.14) | Hoarseness (36.00) | Anxiety | Joint pain* (29.85) | Hoarseness* (40.09) | Shortness of breath | Hair loss (37.74) |
|  |  |  |  | (31.71) |  |  | (36.61) |  |  | (28.07) |  |
|  |  | Headache (39.42) | Itching* | Mouth/  throat sores | Discouraged (34.82) | Heartburn (33.93) | Bloating* (36.11) | Headache | Bloating | Hair loss (28.07) | Numbness & tingling |
|  |  |  | (36.59) | (30.89) |  |  |  | (29.85) | (37.77) |  | (33.96) |
|  |  | Heartburn* (38.44) | General pain (35.43) | Swelling (29.27) | Radiation skin reaction | Discouraged (32.14) | Nausea | Hand-foot syndrome* (29.85) | General pain (37.18) | Concentration (28.07) | Concentration (35.85) |
|  |  |  |  |  | (33.33) |  | (35.71) |  |  |  |  |
|  |  | Mouth/  throat sores | Mouth/  throat sores | Vomiting (28.46) | Mouth/  throat sores | Itching* | Swelling (35.71) | Radiation skin reaction* | Nausea | Nausea | Headache (26.42) |
|  |  | (38.20) | (32.28) |  | (32.14) | (32.00) |  | (28.57) | (35.90) | (26.32) |  |
|  |  | Diarrhea | Blurred vision | Shortness of breath | Cracking at the corners of the mouth | Diarrhea (30.36) | Shortness of breath |  | Urinary frequency* (35.78) | General pain (26.32) | Mouth/  throat sores |
|  |  | (36.50) | (30.89) | (28.46) | (31.53) |  | (33.93) |  |  |  | (26.42) |
|  |  | Hand-foot syndrome (32.36) | Abdominal pain | Cracking at the corners of the mouth | Chills | Cough | Urinary urgency (33.64) |  | Itching | Increased sweating (25.93) |  |
|  |  |  | (30.89) | (27.64) | (31.53) | (30.00) |  |  | (35.19) |  |  |
|  |  | Heart palpitations (31.87) | Heartburn* (30.71) | Muscle pain (26.83) | Urinary urgency (31.53) | Swelling (26.79) | Radiation skin reaction |  | Heartburn* (32.05) | Vaginal dryness (25.00) |  |
|  |  |  |  |  |  |  | (33.33) |  |  |  |  |
|  |  | Vaginal dryness (29.90) | Increased sweating (30.08) | Discouraged (26.02) | Vomiting (27.68) | Shortness of breath | Itching* |  | Chills |  |  |
|  |  |  |  |  |  | (26.79) | (31.48) |  | (30.17) |  |  |
|  |  | Acne | Nosebleed* (28.46) |  | Painful urination (26.13) | Headache (26.79) | Headache (31.25) |  | Increased sweating* (29.18) |  |  |
|  |  | (29.44) |  |  |  |  |  |  |  |  |  |
|  |  |  | Discouraged (27.56) |  | Ringing in ears | Blurred vision | Cracking at the corners of the mouth |  | Blurred vision* (29.18) |  |  |
|  |  |  |  |  | (25.23) | (26.00) | (29.91) |  |  |  |  |
|  |  |  | Fecal incontinence (26.02) |  | Vaginal dryness (25.00) |  | Discouraged (28.57) |  | Diarrhea (29.91) |  |  |
|  |  |  | Hot flashes (25.20) |  |  |  | Ejaculation (27.78) |  | Mouth/  throat sores |  |  |
|  |  |  |  |  |  |  |  |  | (29.06) |  |  |
|  |  |  |  |  |  |  | Increased sweating* (27.78) |  | Wheezing (28.45) |  |  |
|  |  |  |  |  |  |  | Hoarseness (26.17) |  | Muscle pain* (26.92) |  |  |
|  |  |  |  |  |  |  | Heartburn (25.89) |  | Discouraged (26.92) |  |  |
|  |  |  |  |  |  |  | Muscle pain (27.03) |  | Difficulty swallowing* (25.86) |  |  |
|  |  |  |  |  |  |  | Heart palpitations* (25.23) |  |  |  |  |

*Additional items, involved in Korea
