# Supplementary material for: Surveillance of Symptom Burden Using the Patient-Reported Outcome Version of the Common Terminology Criteria for Adverse Events in Patients With Various Types of Cancers During Chemoradiation Therapy: Real-World Study
Source: JMIR Public Health Surveill. 2023 Mar 8;9:e44105. doi: 10.2196/44105 (PMC10034615; doi:10.2196/44105)
Supplement: Multimedia Appendix 2 [file publichealth_v9i1e44105_app2.docx]

| Symptom | Breast  (95% CI) | Colorectal  (95% CI) | Gastric  (95% CI) | Gynecologic  (95% CI) | Head & Neck  (95% CI) | Liver  (95% CI) | Lung (95% CI) | Lymphoma  (95% CI) | Prostate  (95% CI) | Others  (95% CI) |
| --- | --- | --- | --- | --- | --- | --- | --- | --- | --- | --- |
| Oral |  |  |  |  |  |  |  |  |  |  |
| Dry mouth | 0.07  (-0.05, 0.18) | -0.19  (-0.34, -0.04) | 0.18  (0.03, 0.34) | -0.01  (-0.17, 0.15) | 0.33  (0.11, 0.55) | -0.07  (-0.27, 0.13) | -0.09  (-0.21, 0.03) | 0.06  (-0.10, 0.22) | -0.16  (-0.38, 0.07) | -0.08  (-0.31, 0.14) |
| Difficulty Swallowing | - | - | - | - | 0.45  (0.26, 0.65) | - | -0.15  (-0.29, -0.02) | -0.06  (-0.22, 0.09) | - | - |
| Mouth/throat sores | 0.06  (-0.06, 0.17) | 0.08  (-0.07, 0.23) | 0.01  (-0.14, 0.17) | -0.10  (-0.26, 0.07) | 0.42  (0.20, 0.64) | -0.15  (-0.35, 0.05) | -0.03  (-0.15, 0.09) | -0.08  (-0.24, 0.09) | -0.19  (-0.41, 0.04) | -0.06  (-0.29, 0.17) |
| Cracking at the corners of the mouth | - | - | 0.08  (-0.05, 0.21) | -0.08  (-0.23, 0.07) | 0.34 (0.15, 0.53) | - | -0.18 (-0.28, -0.07) | 0.09 (-0.05, 0.22) | - | - |
| Hoarseness | - | - | - | - | 0.05 (-0.17, 0.27) | - | 0.05 (-0.09, 0.20) | -0.10 (-0.26, 0.06) | - | - |
| Gastro-intestinal |  |  |  |  |  |  |  |  |  |  |
| Taste changes | 0.14 (0.02, 0.26) | -0.04 (-0.20, 0.11) | 0.23 (0.07, 0.39) | -0.09 (-0.26, 0.08) | 0.25 (0.02, 0.48) | 0.02 (-0.19, 0.23) | -0.12 (-0.25, 0.00) | -0.14 (-0.31, 0.02) | -0.18 (-0.42, 0.06) | -0.16 (-0.39, 0.08) |
| Decreased appetite | -0.03 (-0.17, 0.11) | -0.04 (-0.22, 0.13) | 0.43 (0.25, 0.61) | 0.00 (-0.19, 0.19) | 0.41 (0.15, 0.67) | 0.13 (-0.10, 0.37) | -0.07 (-0.21, 0.08) | -0.24 (-0.43, -0.05) | -0.39 (-0.65, -0.12) | -0.20 (-0.47, 0.06) |
| Nausea | -0.18 (-0.31, -0.05) | 0.13 (-0.04, 0.29) | 0.19 (0.02, 0.36) | 0.28 (0.10, 0.46) | 0.03 (-0.21, 0.28) | 0.27 (0.04, 0.49) | -0.11 (-0.25, 0.02) | -0.20 (-0.38, -0.02) | -0.11 (-0.37, 0.14) | -0.02 (-0.27, 0.23) |
| Vomiting | -0.12 (-0.21, -0.04) | -0.04 (-0.15, 0.06) | 0.24 (0.13, 0.35) | 0.19 (0.08, 0.30) | 0.01 (-0.15, 0.17) | 0.05 (-0.09, 0.19) | -0.06 (-0.14, 0.02) | -0.12 (-0.23, 0.00) | 0.01 (-0.15, 0.17) | 0.05 (-0.11, 0.21) |
| Heartburn | -0.02 (-0.14, 0.10) | -0.11 (-0.26, 0.05) | -0.05 (-0.21, 0.12) | 0.13 (-0.04, 0.30) | 0.11 (-0.12, 0.34) | 0.03 (-0.17, 0.24) | 0.06 (-0.06, 0.19) | -0.13 (-0.30, 0.04) | - | 0.03 (-0.20, 0.26) |
| Bloating | -0.15 (-0.28, -0.02) | 0.02 (-0.14, 0.18) | 0.15 (-0.02, 0.31) | 0.19 (0.02, 0.37) | - | - | 0.02 (-0.11, 0.15) | -0.07 (-0.25, 0.10) | -0.14 (-0.39, 0.11) | - |
| Hiccups | - | 0.06 (-0.06, 0.18) | - | - | -0.13 (-0.31, 0.05) | - | -0.02 (-0.12, 0.09) | 0.03 (-0.10, 0.16) | - | - |
| Constipation | 0.04 (-0.08, 0.16) | -0.16 (-0.32, 0.00) | -0.19 (-0.35, -0.02) | 0.20 (0.03, 0.37) | 0.19 (-0.05, 0.42) | 0.06 (-0.16, 0.27) | 0.09 (-0.04, 0.21) | -0.13 (-0.30, 0.04) | -0.15 (-0.39, 0.10) | 0.05 (-0.19, 0.29) |
| Diarrhea | -0.06 (-0.15, 0.03) | 0.13 (0.01, 0.25) | 0.23 (0.11, 0.35) | 0.03 (-0.10, 0.16) | -0.03 (-0.20, 0.15) | 0.01 (-0.15, 0.17) | -0.04 (-0.13, 0.05) | -0.21 (-0.33, -0.08) | 0.06 (-0.11, 0.24) | -0.10 (-0.28, 0.08) |
| Abdominal pain | - | 0.14 (-0.01, 0.29) | 0.18 (0.03, 0.34) | 0.04 (-0.14, 0.21) | - | - | -0.16 (-0.28, -0.04) | -0.14 (-0.30, 0.02) | 0.04 (-0.19, 0.28) | - |
| Fecal incontinence | - | 0.14 (0.03, 0.26) | - | 0.07 (-0.07, 0.21) | - | - | -0.14 (-0.24, -0.04) | - | -0.03 (-0.20, 0.15) | - |
| Respiratory |  |  |  |  |  |  |  |  |  |  |
| Shortness of breath | 0.08 (-0.02, 0.18) | -0.24 (-0.36, -0.11) | -0.21 (-0.34, -0.08) | -0.11 (-0.25, 0.03) | -0.04 (-0.23, 0.15) | -0.07 (-0.24, 0.10) | 0.34 (0.25, 0.44) | -0.04 (-0.18, 0.09) | -0.07 (-0.26, 0.12) | -0.06 (-0.25, 0.13) |
| Cough | - | - | - | - | -0.05 (-0.26, 0.17) | - | 0.26 (0.12, 0.39) | -0.20 (-0.37, -0.04) | -0.26 (-0.47, -0.04) | - |
| Wheezing | - | - | - | - | -0.09 (-0.26, 0.09) | - | 0.16 (0.06, 0.27) | -0.13 (-0.26, 0.01) | -0.11 (-0.29, 0.06) | - |
| Cardio  /circulatory |  |  |  |  |  |  |  |  |  |  |
| Swelling | 0.26 (0.13, 0.39) | -0.28 (-0.45, -0.12) | -0.06 (-0.23, 0.11) | -0.10 (-0.28, 0.08) | 0.03 (-0.21, 0.27) | 0.04 (-0.18, 0.26) | -0.09 (-0.22, 0.04) | 0.20 (0.03, 0.38) | 0.06 (-0.19, 0.30) | -0.24 (-0.48, 0.01) |
| Heart palpitations | -0.01 (-0.15, 0.12) | - | - | - | - | - | -0.03 (-0.15, 0.08) | 0.03 (-0.11, 0.17) | 0.07 (-0.13, 0.26) | - |
| Cutaneous |  |  |  |  |  |  |  |  |  |  |
| Skin dryness | 0.11 (-0.01, 0.24) | 0.01 (-0.15, 0.16) | 0.03 (-0.13, 0.19) | -0.08 (-0.24, 0.08) | - | - | -0.11 (-0.23, 0.02) | 0.01 (-0.16, 0.17) | - | - |
| Acne | 0.09 (-0.02, 0.20) | 0.06 (-0.05, 0.18) | - | - | -0.14 (-0.32, 0.03) | - | -0.03 (-0.13, 0.07) | -0.08 (-0.20, 0.05) | - | - |
| Hair loss | 0.07 (-0.05, 0.19) | -0.06 (-0.21, 0.09) | 0.02 (-0.14, 0.17) | 0.01 (-0.15, 0.17) | 0.17 (-0.05, 0.40) | 0.04 (-0.16, 0.24) | 0.03 (-0.09, 0.15) | -0.19 (-0.35, -0.03) | -0.13 (-0.36, 0.10) | -0.04 (-0.26, 0.19) |
| Itching | 0.10 (-0.01, 0.21) | 0.05 (-0.09, 0.19) | -0.04 (-0.18, 0.10) | -0.13 (-0.28, 0.02) | -0.01 (-0.22, 0.21) | - | -0.01 (-0.12, 0.11) | -0.06 (-0.21, 0.10) | - | - |
| Hand-foot syndrome | 0.19 (0.09, 0.28) | -0.01 (-0.13, 0.12) | 0.17 (0.04, 0.30) | -0.21 (-0.34, -0.07) | 0.04 (-0.15, 0.22) | 0.21 (0.04, 0.38) | -0.15 (-0.25, -0.05) | -0.13 (-0.27, 0.00) | - | -0.17 (-0.36, 0.02) |
| Radiation skin reaction | 0.44 (0.12, 0.75) | - | - | -0.52 (-0.95, -0.09) | 0.03 (-0.31, 0.37) | -0.43 (-1.09, 0.23) | 0.11 (-0.23, 0.45) | -0.31 (-1.31, 0.70) | - | -0.28 (-0.73, 0.16) |
| Neurological |  |  |  |  |  |  |  |  |  |  |
| Numbness & tingling | 0.11 (-0.02, 0.25) | 0.08 (-0.09, 0.26) | 0.12 (-0.06, 0.31) | 0.14 (-0.06, 0.33) | -0.10 (-0.36, 0.17) | 0.1 (-0.14, 0.34) | -0.24 (-0.38, -0.10) | 0.08 (-0.11, 0.27) | -0.20 (-0.46, 0.07) | -0.27 (-0.54, 0.00) |
| Dizziness | -0.10 (-0.21, 0.01) | 0.04 (-0.09, 0.18) | - | 0.08 (-0.06, 0.23) | 0.22 (0.01, 0.43) | - | 0.02 (-0.09, 0.13) | -0.03 (-0.18, 0.12) | -0.16 (-0.37, 0.05) | - |
| Visual  /perceptual |  |  |  |  |  |  |  |  |  |  |
| Blurred vision | 0.19 (0.05, 0.34) | -0.10 (-0.25, 0.05) | - | - | 0.01 (-0.21, 0.24) | - | -0.09 (-0.22, 0.05) | - | - | - |
| Watery eyes | 0.26 (0.13, 0.40) | -0.14 (-0.28, 0.00) | - | - | 0.11 (-0.09, 0.32) | - | -0.15 (-0.27, -0.03) | - | - | - |
| Ringing in ears | - | - | - | -0.16 (-0.33, 0.01) | 0.10 (-0.09, 0.29) | - | 0.03 (-0.10, 0.15) | - | 0.04 (-0.15, 0.23) | - |
| Attention  /memory |  |  |  |  |  |  |  |  |  |  |
| Concentration | 0.11 (0.01, 0.21) | -0.09 (-0.22, 0.04) | 0.04 (-0.09, 0.18) | -0.09 (-0.23, 0.05) | 0.06 (-0.13, 0.25) | 0.14 (-0.03, 0.32) | -0.02 (-0.12, 0.08) | -0.07 (-0.21, 0.07) | -0.05 (-0.25, 0.14) | -0.11 (-0.30, 0.09) |
| Memory | 0.24 (0.09, 0.39) | -0.11 (-0.26, 0.04) | - | - | - | - | -0.10 (-0.23, 0.03) | - | 0.00 (-0.22, 0.23) | - |
| Pain |  |  |  |  |  |  |  |  |  |  |
| General pain | 0.20 (0.06, 0.33) | -0.15 (-0.33, 0.03) | -0.17 (-0.36, 0.01) | -0.11 (-0.3, 0.09) | 0.24 (-0.02, 0.51) | 0.16 (-0.08, 0.40) | -0.01 (-0.15, 0.14) | -0.09 (-0.28, 0.10) | -0.19 (-0.46, 0.08) | 0.10 (-0.17, 0.37) |
| Headache | 0.05 (-0.06, 0.16) | -0.08 (-0.22, 0.05) | -0.17 (-0.32, -0.03) | 0.02 (-0.13, 0.17) | 0.06 (-0.15, 0.26) | 0.14 (-0.04, 0.32) | -0.03 (-0.14, 0.08) | 0.08 (-0.07, 0.22) | 0.10 (-0.11, 0.30) | -0.05 (-0.26, 0.15) |
| Muscle pain | 0.32 (0.18, 0.46) | - | -0.15 (-0.33, 0.03) | -0.13 (-0.31, 0.06) | - | 0.13 (-0.10, 0.36) | -0.08 (-0.22, 0.06) | -0.10 (-0.29, 0.08) | -0.04 (-0.30, 0.22) | -0.20 (-0.46, 0.05) |
| Joint pain | 0.27 (0.15, 0.40) | -0.15 (-0.31, 0.01) | -0.15 (-0.32, 0.02) | -0.18 (-0.35, 0.00) | - | 0.13 (-0.09, 0.34) | -0.01 (-0.14, 0.12) | -0.09 (-0.26, 0.09) | 0.02 (-0.23, 0.27) | 0.01 (-0.24, 0.25) |
| Sleep/wake |  |  |  |  |  |  |  |  |  |  |
| Insomnia | 0.07 (-0.07, 0.21) | -0.18 (-0.37, 0.01) | -0.02 (-0.21, 0.18) | 0.04 (-0.16, 0.24) | 0.20 (-0.08, 0.48) | 0.17 (-0.08, 0.42) | 0.02 (-0.13, 0.17) | -0.03 (-0.24, 0.17) | -0.05 (-0.34, 0.23) | -0.27 (-0.55, 0.01) |
| Fatigue | 0.06 (-0.07, 0.18) | -0.21 (-0.38, -0.05) | 0.09 (-0.08, 0.26) | -0.01 (-0.19, 0.17) | 0.32 (0.08, 0.56) | 0.26 (0.04, 0.48) | -0.09 (-0.22, 0.05) | -0.08 (-0.25, 0.10) | -0.07 (-0.32, 0.18) | -0.05 (-0.30, 0.19) |
| Mood |  |  |  |  |  |  |  |  |  |  |
| Anxious | 0.03 (-0.09, 0.15) | -0.13 (-0.28, 0.03) | -0.02 (-0.19, 0.14) | -0.02 (-0.18, 0.15) | 0.04 (-0.19, 0.26) | 0.28 (0.07, 0.49) | 0.05 (-0.07, 0.18) | -0.19 (-0.35, -0.02) | 0.02 (-0.21, 0.26) | 0.02 (-0.21, 0.25) |
| Discouraged | 0.07 (-0.03, 0.17) | -0.05 (-0.18, 0.08) | -0.10 (-0.23, 0.03) | -0.03 (-0.17, 0.11) | 0.12 (-0.07, 0.31) | 0.07 (-0.10, 0.25) | 0.02 (-0.09, 0.12) | -0.06 (-0.20, 0.07) | 0.03 (-0.17, 0.22) | -0.06 (-0.26, 0.13) |
| Sad | 0.06 (-0.05, 0.18) | -0.16 (-0.30, -0.01) | -0.06 (-0.22, 0.09) | 0.01 (-0.15, 0.18) | 0.08 (-0.14, 0.30) | 0.11 (-0.09, 0.31) | 0.02 (-0.10, 0.14) | -0.16 (-0.32, 0.00) | 0.19 (-0.04, 0.41) | 0.03 (-0.19, 0.26) |
| Gynecological |  |  |  |  |  |  |  |  |  |  |
| Vaginal discharge | 0.00 (-0.09, 0.09) | - | - | 0.03 (-0.07, 0.13) | - | - | - | -0.08 (-0.23, 0.08) | - | - |
| Vaginal dryness | 0.15 (0.02, 0.28) | - | - | -0.10 (-0.24, 0.05) | - | - | - | -0.19 (-0.42, 0.03) | -0.12 (-0.61, 0.38) | - |
| Urinary |  |  |  |  |  |  |  |  |  |  |
| Painful urination | - | - | - | 0.27 (0.13, 0.41) | - | - | -0.15 (-0.25, -0.04) | -0.13 (-0.26, -0.01) | 0.27 (0.10, 0.44) | - |
| Urinary urgency | - | - | - | 0.05 (-0.11, 0.21) | - | - | -0.16 (-0.28, -0.05) | 0.07 (-0.07, 0.21) | 0.26 (0.06, 0.45) | - |
| Urinary frequency | - | - | - | -0.01 (-0.20, 0.19) | - | - | -0.16 (-0.30, -0.02) | 0.12 (-0.05, 0.30) | 0.24 (0.00, 0.48) | - |
| Urinary incontinence | - | - | - | 0.01 (-0.12, 0.14) | - | - | -0.08 (-0.17, 0.02) | 0.02 (-0.09, 0.14) | 0.17 (0.01, 0.33) | - |
| Sexual |  |  |  |  |  |  |  |  |  |  |
| Achieve and maintain erection | - | -0.28 (-0.84, 0.27) | - | - | - | - | - | 0.10 (-0.48, 0.68) | 0.50 (-0.41, 1.40) | - |
| Ejaculation | - | - | - | - | - | - | - | NA | NA | - |
| Decreased libido | 0.20 (-0.11, 0.51) | -0.04 (-0.40, 0.32) | - | -0.26 (-0.68, 0.17) | - | - | 0.00 (-0.33, 0.34) | - | -1.46 (-3.50, 0.57) | - |
| Pain with sexual intercourse | 0.14 (-0.35, 0.63) | - | - | -0.02 (-0.62, 0.57) | - | - | - | -0.34 (-1.16, 0.47) | NA | - |
| Miscellaneous |  |  |  |  |  |  |  |  |  |  |
| Breast swelling and tenderness | - | -0.06 (-0.16, 0.03) | - | - | - | - | - | - | 0.06 (-0.03, 0.16) | - |
| Chills | - | -0.08 (-0.23, 0.08) | 0.02 (-0.13, 0.18) | -0.06 (-0.23, 0.12) | - | - | 0.13 (0.00, 0.26) | -0.10 (-0.26, 0.06) | - | - |
| Increased sweating | 0.20 (0.06, 0.34) | -0.07 (-0.24, 0.11) | - | -0.03 (-0.21, 0.16) | - | - | -0.08 (-0.23, 0.07) | -0.13 (-0.32, 0.06) | 0.03 (-0.24, 0.30) | - |
| Hot flashes | 0.19 (0.06, 0.32) | -0.08 (-0.23, 0.08) | - | -0.12 (-0.28, 0.03) | - | - | - | -0.13 (-0.30, 0.05) | 0.07 (-0.18, 0.31) | - |
| Nosebleeds | - | 0.23 (0.12, 0.33) | - | - | -0.03 (-0.18, 0.13) | - | -0.05 (-0.14, 0.04) | -0.17 (-0.29, -0.06) | - | - |
| Body odor | - | 0.07 (-0.02, 0.16) | - | 0.07 (-0.04, 0.18) | - | - | - | -0.14 (-0.24, -0.04) | - | - |

Adjusted age, sex, ECOG and treatment types

Bold with grey backgroud indicates statistically significant more severe symptoms than other type of cancer. (P < 0.05)
