# Supplementary material for: Surveillance of Symptom Burden Using the Patient-Reported Outcome Version of the Common Terminology Criteria for Adverse Events in Patients With Various Types of Cancers During Chemoradiation Therapy: Real-World Study
Source: JMIR Public Health Surveill. 2023 Mar 8;9:e44105. doi: 10.2196/44105 (PMC10034615; doi:10.2196/44105)
Supplement: Multimedia Appendix 3 [file publichealth_v9i1e44105_app3.docx]

**Multimedia Appendix 3**

Association between symptoms and health-related quality of life in different types of cancer.^a,b^

| Symptom | | Overall, coefficient (95% CI) | Breast, coefficient (95% CI) | Colorectal, coefficient (95% CI) | Gynecologic, coefficient (95% CI) | Gastric, coefficient (95% CI) | Head and neck/esophageal, coefficient (95% CI) | Prostate/bladder, coefficient (95% CI) | Liver, coefficient (95% CI) | Lung, coefficient (95% CI) | Lymphoma/myeloma (95% CI) | Others (95% CI) |
| --- | --- | --- | --- | --- | --- | --- | --- | --- | --- | --- | --- | --- |
| **Oral** | |  |  |  |  |  |  |  |  |  |  |  |
|  | Dry mouth | *–4.39 (–5.74 to –3.03)* | *–6.40 (–8.14 to –4.65)* | *–5.83 (–8.89 to –2.78)* | *–6.55 (–9.42 to –3.69)* | *–6.14 (–9.11 to –3.17)* | –3.75 (–9.15 to 1.66) | –3.65 (–9.30 to 2.01) | *–8.75 (–12.55 to –4.95)* | *–3.76 (–6.00 to –1.52)* | *–5.09 (–8.11 to –2.08)* | *–4.74 (–9.34 to –0.14)* |
|  | Difficulty Swallowing | *–6.1 (–9.16 to –3.04)* |  |  |  |  | *–5.48 (–10.71 to –0.24)* |  |  | *–4.88 (–7.61 to –2.14)* | *–5.65 (–10.96 to –0.34)* |  |
|  | Mouth/throat sores | *–3.75 (–5.1 to –2.39)* | *–4.87 (–6.67 to –3.08)* | *–5.18 (–7.62 to –2.74)* | –1.69 (–4.98 to 1.60) | *–3.69 (–7.01 to –0.38)* | *–5.13 (–9.02 to –1.24)* | *–7.45 (–13.87 to –1.03)* | *–6.86 (–12.71 to –1.00)* | –1.39 (–3.60 to 0.81) | *–4.05 (–7.54 to –0.56)* | 2.47 (–3.33 to 8.27) |
|  | Cracking at the corners of the mouth | *–2.67 (–5.2 to –0.13)* |  |  | *–7.38 (–11.71 to –3.05)* | *–5.28 (–9.17 to –1.39)* | *–9.9 (–16.46 to –3.34)* |  |  | –1.62 (–4.84 to 1.60) | *–5.06 (–9.32 to –0.81)* |  |
|  | Hoarseness | *–4.65 (–7.53 to –1.77)* |  |  |  |  | *–6.36 (–11.77 to –0.95)* |  |  | *–5.75 (–8.13 to –3.38)* | *–7.31 (–11.48 to –3.15)* |  |
| **Gastrointestinal** | |  |  |  |  |  |  |  |  |  |  |  |
|  | Taste changes | *–5.98 (–7.25 to –4.72)* | *–7.97 (–9.57 to –6.38)* | *–6.98 (–9.62 to –4.34)* | *–7.83 (–10.41 to –5.25)* | *–9.05 (–12.04 to –6.07)* | *–5.29 (–10.18 to –0.41)* | *–7.15 (–11.48 to –2.83)* | *–8.57 (–12.71 to –4.44)* | *–5.54 (–7.42 to –3.65)* | *–4.80 (–7.99 to –1.61)* | –3.46 (–8.25 to 1.33) |
|  | Decreased appetite | *–6.35 (–7.47 to –5.24)* | *–7.88 (–9.34 to –6.41)* | *–8.45 (–10.93 to –5.98)* | –7.28 (–9.44 to –5.13) | *–7.9 (–10.36 to –5.44)* | *–5.09 (–9.17 to –1.00)* | *–6.06 (–10.31 to –1.8)* | *–9.74 (–12.72 to –6.75)* | *–5.2 (–6.81 to –3.59)* | *–5.76 (–8.72 to –2.79)* | *–8.27 (–12.21 to –4.34)* |
|  | Nausea | *–3.96 (–5.17 to –2.75)* | *–7.06 (–8.75 to –5.37)* | *–7.51 (–9.64 to –5.38)* | *–4.56 (–6.87 to –2.24)* | *–3.49 (–6.23 to –0.76)* | *–11.46 (–16.28 to –6.65)* | –3.77 (–8.87 to 1.33) | *–7.23 (–10.83 to –3.62)* | *–5.13 (–6.94 to –3.32)* | *–4.17 (–7.42 to –0.91)* | *–5.28 (–9.42 to –1.13)* |
|  | Vomiting | *–5.64 (–7.56 to –3.73)* | *–9.07 (–12.04 to –6.10)* | –10.11 (–14.46 to –5.77) | *–7.46 (–11.06 to –3.85)* | *–4.03 (–7.34 to –0.73)* | *–8.44 (–15.61 to –1.28)* | –4.71 (–13.36 to 3.94) | *–5.27 (–11.92 to 1.38)* | *–5.63 (–8.89 to –2.38)* | 1.24 (–4.15 to 6.64) | *–8.81 (–14.44 to –3.19)* |
|  | Heartburn | *–5.19 (–6.51 to –3.87)* | *–6.47 (–8.17 to –4.77)* | *–6.44 (–9.49 to –3.39)* | *–5.32 (–7.83 to –2.80)* | *–5.4 (–8.73 to –2.08)* | *–9.17 (–14.09 to –4.25)* |  | *–5.41 (–10.62 to –0.19)* | *–5.02 (–6.79 to –3.26)* | *–7 (–10.05 to –3.95)* | –4.93 (–10.01 to 0.16) |
|  | Bloating | *–4.62 (–6 to –3.25)* | *–6.77 (–8.40 to –5.14)* | *–5.42 (–8.16 to –2.68)* | *–4.91 (–7.53 to –2.30)* | *–6.98 (–9.62 to –4.35)* |  | *–10.5 (–16.57 to –4.44)* |  | *–2.67 (–4.64 to –0.69)* | *–5.27 (–8.25 to –2.29)* |  |
|  | Hiccups | *–3.25 (–6.22 to –0.29)* |  | *–6.75 (–10.09 to –3.41)* |  |  | *–14.73 (–25.35 to –4.1)* |  |  | *–3.6 (–6.57 to –0.63)* | *–6.03 (–10.69 to –1.36)* |  |
|  | Constipation | *–3.55 (–4.82 to –2.28)* | *–7.49 (–9.07 to –5.92)* | *–4.93 (–7.98 to –1.88)* | *–4.5 (–7.39 to –1.62)* | *–7.78 (–11.00 to –4.56)* | –3.43 (–7.67 to 0.82) | –2.01 (–6.84 to 2.81) | *–8.53 (–12.20 to –4.85)* | *–3.83 (–5.65 to –2.01)* | *–6.55 (–9.63 to –3.47)* | *–6.15 (–10.61 to –1.69)* |
|  | Diarrhea | *–1.8 (–3.54 to –0.07)* | *–6.34 (–8.66 to –4.02)* | *–9.57 (–12.42 to –6.73)* | *–5.59 (–9.43 to –1.76)* | *–4.81 (–8.44 to –1.17)* | –3.24 (–10.83 to 4.36) | *–6.21 (–11.52 to –0.90)* | –3.09 (–10.06 to 3.88) | –2.83 (–5.77 to 0.10) | *–6.83 (–12.07 to –1.58)* | *–8.22 (–15.27 to –1.16)* |
|  | Abdominal pain | *–5.48 (–7.39 to –3.57)* |  | *–7.67 (–10.14 to –5.21)* | *–7.65 (–10.45 to –4.86)* | *–4.46 (–7.84 to –1.07)* |  | *–11.53 (–17.85 to –5.21)* |  | *–5.45 (–7.92 to –2.98)* | *–6.09 (–9.73 to –2.44)* |  |
|  | Fecal incontinence | *–4.39 (–7.64 to –1.14)* |  | *–7.65 (–11.28 to –4.01)* | *–5.60 (–9.64 to –1.56)* |  |  | *–12.45 (–20.19 to –4.70)* |  | *–6.33 (–9.99 to –2.68)* |  |  |
| **Respiratory** | |  |  |  |  |  |  |  |  |  |  |  |
|  | Shortness of breath | *–6.44 (–8.03 to –4.86)* | *–11.35 (–13.21 to –9.50)* | *–10.41 (–15.36 to –5.46)* | *–7.16 (–11.10 to –3.21)* | *–8.49 (–13.03 to –3.96)* | *–12.38 (–18.66 to –6.1)* | *–12.7 (–18.18 to –7.21)* | –6.71 (–13.51 to 0.10) | *–5.82 (–7.75 to –3.89)* | *–8.58 (–11.93 to –5.22)* | *–12.32 (–19.27 to –5.37)* |
|  | Cough | *–3.28 (–5.97 to –0.6)* |  |  |  |  | –3.43 (–9.01 to 2.15) | *–10.86 (–18.44 to –3.27)* |  | *–6.16 (–8.36 to –3.95)* | –0.85 (–5.35 to 3.65) |  |
|  | Wheezing | *–6.59 (–9.86 to –3.31)* |  |  |  |  | –7.15 (–15.67 to 1.37) | *–11.93 (–19.56 to –4.30)* |  | *–4.69 (–7.36 to –2.02)* | *–6.75 (–12.13 to –1.37)* |  |
| **Cardio/circulatory** | |  |  |  |  |  |  |  |  |  |  |  |
|  | Swelling | *–4.05 (–5.28 to –2.81)* | *–5.7 (–7.07 to –4.33)* | *–7.12 (–10.66 to –3.58)* | *–4.27 (–6.98 to –1.56)* | *–8.38 (–11.34 to –5.43)* | –3.36 (–9.58 to 2.86) | *–5.13 (–9.93 to –0.34)* | *–7.02 (–11.82 to –2.23)* | *–2.59 (–4.95 to –0.23)* | *–5.29 (–7.78 to –2.81)* | –4.28 (–10.81 to 2.25) |
|  | Heart palpitations | *–5.43 (–7.68 to –3.18)* | *–9.27 (–11.26 to –7.28)* |  |  |  |  | *–9.55 (–15.61 to –3.49)* |  | *–5.55 (–8.32 to –2.77)* | *–8.04 (–12.25 to –3.84)* |  |
| **Cutaneous** | |  |  |  |  |  |  |  |  |  |  |  |
|  | Skin dryness | *–5.02 (–6.53 to –3.51)* | *–6.11 (–7.85 to –4.37)* | *–6.59 (–9.28 to –3.90)* | *–5.70(–9.00 to –2.40)* | *–4.85 (–8.33 to –1.37)* |  |  |  | *–5.16 (–7.30–3.02)* | *–6.61 (–9.73 to –3.50)* |  |
|  | Acne | *–2.93 (–5.19 to –0.68)* | *–5.57 (–7.85 to –3.29)* | *–3.81 (–7.33 to –0.30)* |  |  | –5.45 (–17.54 to 6.65) |  |  | *–5.27 (–8.63 to –1.90* | *–8.02 (–12.88 to –3.15)* |  |
|  | Hair loss | *–3.32 (–4.64 to –1.99)* | *–4.87 (–6.39 to –3.35)* | *–6.54 (–9.76 to –3.32)* | –2.10(–5.28 to 1.08) | *–5.91 (–9.29 to –2.53)* | *–5.78 (–10.49 to –1.07)* | –4.89 (–12.78 to 3.01) | *–5.83 (–10.22 to –1.45)* | *–3.94 (–6.13 to –1.74)* | 0.25 (–3.31 to 3.82) | *–5.33 (–10.11 to –0.54)* |
|  | Itching | *–4.71 (–6.32 to –3.1)* | *–6.54 (–8.44 to –4.64)* | *–4.88 (–7.8 to –1.96)* | *–4.14 (–7.62 to –0.66)* | –2.8 (–7.01 to 1.42) | *–6.45 (–12.32 to –0.59)* |  |  | *–5.14 (–7.49 to –2.80)* | *–5.93 (–9.61 to –2.26)* |  |
|  | Hand-foot syndrome | *–1.98 (–3.66 to –0.3)* | *–5.94 (–7.83 to –4.05)* | *–4.85 (–8.43 to –1.28)* | *–4.91 (–9.69 to –0.13)* | –0.43 (–4.33 to 3.48) | *–8.68 (–15.48 to –1.89)* |  | –3.55 (–8.10 to 1.00) | –2.27 (–5.39 to 0.86) | *–5.3 (–9.69 to –0.91)* | 0.14 (–9.30 to 9.58) |
|  | Radiation skin reaction | *–1.61 (–4.73 to 1.51)* | *–4.43 (–7.88 to –0.99)* |  | 3.33 (–13.7 to 20.37) |  | –3.15 (–9.69 to 3.39) |  | –16.36 (–159.07 to 126.36) | *–5.08 (–9.66 to –0.49)* | NA | 3.73 (–4.56 to 12.02) |
| **Neurological** | |  |  |  |  |  |  |  |  |  |  |  |
|  | Numbness and tingling | *–3.88 (–5.02 to –2.75)* | *–6.64 (–7.96 to –5.31)* | *–5.5 (–7.77 to –3.24)* | *–5.24 (–7.83 to –2.65)* | *–7.02 (–9.64 to –4.41)* | *–4.89 (–9.70 to –0.08)* | *–5.26 (–9.96 to –0.57)* | *–6.91 (–10.73 to –3.1)* | *–3.16 (–5.12 to –1.2)* | *–5.32 (–7.80 to –2.83)* | *–5.65 (–9.86 to –1.44)* |
|  | Dizziness | *–7.24 (–8.92 to –5.55)* | *–10.56 (–12.41 to –8.71)* | *–9.98 (–13.02 to –6.93)* | *–8.33 (–11.49 to –5.17)* |  | *–9.28 (–15.25 to –3.31)* | *–11.07 (–17.22 to –4.92)* |  | *–6.12 (–8.32 to –3.92)* | *–11.34 (–14.65 to –8.03)* |  |
| **Visual/perceptual** | |  |  |  |  |  |  |  |  |  |  |  |
|  | Blurred vision | *–3.95 (–5.82 to –2.09)* | –6.79 (–8.44 to –5.15) | *–8.75 (–12.42 to –5.08)* |  |  | *–7.09 (–12.74 to –1.44)* |  |  | *–5.34 (–7.94 to –2.75)* |  |  |
|  | Watery eyes | *–2.43 (–4.49 to –0.38)* | *–5.78 (–7.57 to –3.99)* | *–7.06 (–11.58 to –2.55)* |  |  | *–7.33 (–13.74 to –0.93)* |  |  | –1.59 (–4.83 to 1.66) |  |  |
|  | Ringing in ears | *–2.78 (–5.9 to 0.34)* |  |  | –0.76 (–4.73 to 3.21) |  | –6.61 (–14.07 to 0.84) | *–12.99 (–20.41 to –5.57)* |  | *–2.99 (–5.91 to –0.07)* |  |  |
| **Attention/memory** | |  |  |  |  |  |  |  |  |  |  |  |
|  | Concentration | *–7.54 (–9.06 to –6.01)* | *–9.91 (–11.61 to –8.21)* | *–10.32 (–13.76 to –6.87)* | *–4.5 (–7.87 to –1.12)* | *–8.59 (–12.22 to –4.97)* | *–11.29 (–16.91 to –5.67)* | *–14.12 (–18.33 to –9.91)* | *–10.67 (–15.52 to –5.81)* | *–8.22 (–10.67 to –5.77)* | *–9.70 (–13.25 to –6.14)* | *–10.57 (–16.98 to –4.15)* |
|  | Memory | *–9.23 (–11.06 to –7.41)* | *–9.79 (–11.42 to –8.15)* | *–10.4 (–13.66 to –7.15)* |  |  |  | *–12.09 (–16.67 to –7.52)* |  | *–7.48 (–9.70 to –5.26)* |  |  |
| **Pain** | |  |  |  |  |  |  |  |  |  |  |  |
|  | General pain | *–6.1 (–7.2 to –5)* | *–7.26 (–8.47 to –6.06)* | *–7.99 (–10.30 to –5.68)* | *–5.95 (–8.65 to –3.24)* | *–8.79 (–11.46 to –6.11)* | *–7.78 (–11.29 to –4.27)* | *–8.58 (–13.50 to –3.66)* | *–7.39 (–11.04 to –3.75)* | *–7.14 (–8.73 to –5.56)* | *–5.73 (–8.49 to –2.97)* | *–7.27 (–10.6 to –3.94)* |
|  | Headache | *–5.33 (–6.8 to –3.86)* | *–6.43 (–8.14 to –4.72)* | *–7.16 (–10.76 to –3.56)* | *–5.81 (–9.20 to –2.42)* | *–7.36 (–12.06 to –2.67)* | *–6.40 (–11.99 to –0.81)* | *–6.23 (–10.92 to –1.55)* | *–7.27 (–11.74 to –2.80)* | *–6.66 (–9.07 to –4.25)* | *–8.12 (–10.84 to –5.40)* | *–7.32 (–14.29 to –0.36)* |
|  | Muscle pain | *–4.81 (–6.11 to –3.52)* | *–6.65 (–7.93 to –5.37)* |  | *–4.82 (–7.49 to –2.16)* | *–6.98 (–10.54 to –3.42)* |  | *–5.88 (–11.15 to –0.61)* | *–7.56 (–12.5 to –2.61)* | *–4.99 (–6.92 to –3.05)* | *–7.56 (–10.33 to –4.79)* | *–6.76 (–11.98 to –1.55)* |
|  | Joint pain | *–4.98 (–6.25 to –3.71)* | *–6.95 (–8.19 to –5.70)* | *–7.40 (–10.49 to –4.31)* | *–4.53 (–7.47 to –1.59)* | *–5.03 (–9.16 to –0.91)* |  | *–8.72 (–14.98 to –2.46)* | *–6.97 (–11.32 to –2.62)* | *–3.96 (–6.05 to –1.87)* | *–6.03 (–9.17 to –2.90)* | *–6.19 (–10.51 to –1.87)* |
| **Sleep/wake** | |  |  |  |  |  |  |  |  |  |  |  |
|  | Insomnia | *–4.07 (–5.15 to –3)* | *–7.67 (–8.92 to –6.43)* | *–6.70 (–8.73 to –4.67)* | *–4.57 (–7.01 to –2.14)* | *–7.29 (–10.13 to –4.45)* | *–9.21 (–12.39 to –6.04)* | *–4.05 (–7.8 to –0.3)* | *–8 (–11.18 to –4.82)* | *–4.8 (–6.34 to –3.26)* | *–4.20 (–6.61 to –1.79)* | *–6.91 (–11.17 to –2.65)* |
|  | Fatigue | *–8.15 (–9.32 to –6.97)* | *–9.96 (–11.31 to –8.62)* | *–10.53 (–12.66 to –8.4)* | *–9.34 (–11.99 to –6.69)* | *–11.36 (–13.67 to –9.05)* | *–9.01 (–12.27 to –5.76)* | *–9.08 (–13.76 to –4.4)* | *–13.01 (–16.4 to –9.62)* | *–8.56 (–10.21 to –6.91)* | *–7.85 (–10.23 to –5.47)* | *–9.95 (–13.42 to –6.48)* |
| **Mood** | |  |  |  |  |  |  |  |  |  |  |  |
|  | Anxious | *–8.22 (–9.48 to –6.96)* | *–8.11 (–9.61 to –6.62)* | *–8.08 (–10.91 to –5.26)* | *–7.01 (–9.93 to –4.08)* | *–9.33 (–12.46 to –6.2)* | *–10.81 (–15.62 to –6.01)* | *–5.87 (–10.36 to –1.39)* | *–9.7 (–13.67 to –5.74)* | *–6.49 (–8.3 to –4.69)* | *–8.06 (–11.43 to –4.69)* | *–11.49 (–15.47 to –7.52)* |
|  | Discouraged | *–6.18 (–7.74 to –4.61)* | *–8.49 (–10.21 to –6.76)* | *–7.46 (–10.87 to –4.05)* | *–5.32 (–8.57 to –2.06)* | *–12.74 (–16.96 to –8.51)* | *–14.56 (–20.27 to –8.85)* | *–7.39 (–13.97 to –0.81)* | *–11.4 (–17.22 to –5.58)* | *–5.71 (–8.16 to –3.27)* | *–5.77 (–10.13 to –1.42)* | *–9.77 (–16.13 to –3.4)* |
|  | Sad | *–8.71 (–10 to –7.41)* | *–8.62 (–10.11 to –7.13)* | *–8.38 (–11.65 to –5.12)* | *–6.81 (–9.78 to –3.84)* | *–6.05 (–9.46 to –2.65)* | *–10.09 (–15.12 to –5.06)* | *–6.96 (–11.34 to –2.57)* | *–11.04 (–15.01 to –7.07)* | *–7.38 (–9.31 to –5.44)* | *–10 (–13.39 to –6.61)* | *–10.57 (–15.21 to –5.92)* |
| **Gynecological** | |  |  |  |  |  |  |  |  |  |  |  |
|  | Vaginal discharge | *–3.03 (–6.93 to 0.87)* | –1.75 (–4.87 to 1.37) |  | *–6.64 (–13.18 to –0.1)* |  |  |  |  |  | 1.34 (–12.25 to 14.93) |  |
|  | Vaginal dryness | *–1.35 (–3.95 to 1.24)* | *–4.7 (–6.71 to –2.69)* |  | –2.98 (–7.37 to 1.41) |  |  | –11.96 (–76.1 to 52.18) |  |  | –2.9 (–16.13 to 10.32) |  |
| **Urinary** | |  |  |  |  |  |  |  |  |  |  |  |
|  | Painful urination | *–5.08 (–8.39 to –1.77)* |  |  | *–3.95 (–7.64 to –0.25)* |  |  | 1.67 (–4.35 to 7.69) |  | *–7.38 (–11.01 to –3.76)* | *–9.04 (–16.06 to –2.02)* |  |
|  | Urinary urgency | *–4.52 (–7.46 to –1.58)* |  |  | *–6.74 (–10.58 to –2.9)* |  |  | –5.03 (–10.42 to 0.36) |  | *–5.34 (–8.34 to –2.33)* | *–5.19 (–9.78 to –0.6)* |  |
|  | Urinary frequency | *–0.5 (–2.91 to 1.9)* |  |  | –1.35 (–5.07 to 2.38) |  |  | –2.4 (–7.18 to 2.39) |  | *–4.3 (–6.6 to –1.99)* | –3.12 (–6.43 to 0.2) |  |
|  | Urinary incontinence | *–5.07 (–8.68 to –1.47)* |  |  | *–4.64 (–9.26 to –0.01)* |  |  | *–5.4 (–10.56 to –0.24)* |  | *–7.32 (–11.12 to –3.52)* | –*7.57 (–15.04 to –0.09)* |  |
| **Sexual** | |  |  |  |  |  |  |  |  |  |  |  |
|  | Achieve and maintain erection | *–8.07 (–14.52 to –1.61)* |  | *–6.86 (–10.93 to –2.78)* |  |  |  | –11.23 (–11.68 to –10.78) |  |  | –4.51 (–10.18 to 1.17) |  |
|  | Ejaculation | *–24.49 (–41.65 to –7.33)* |  |  |  |  |  |  |  |  | –2.63 (–10.83 to 5.56) |  |
|  | Decreased libido | *–4.27 (–6.73 to –1.81)* | *–6.27 (–8.52 to –4.02)* | *–4.58 (–7.73 to –1.43)* | *–7.13 (–13.16 to –1.09)* |  |  | NA |  | *–6.24 (–9.03 to –3.45)* |  |  |
|  | Pain with sexual intercourse | *–1.96 (–6.74 to 2.81)* | *–5 (–8.4 to –1.59)* |  | –7.4 (–22.56 to 7.76) |  |  |  |  |  | –20.06 (–72.18 to 32.06) |  |
| **Miscellaneous** | |  |  |  |  |  |  |  |  |  |  |  |
|  | Breast swelling and tenderness | *–0.24 (–13.67 to 13.18)* |  | 3.79 (–8.21 to 15.78) |  |  |  | –0.59 (–14.81 to 13.63) |  |  |  |  |
|  | Chills | *–6.3 (–8.24 to –4.35)* |  | *–9.83 (–12.72 to –6.93)* | *–6.87 (–10.13 to –3.61)* | *–7.91 (–11.11 to –4.7)* |  |  |  | *–6.27 (–8.25 to –4.3)* | *–10.35 (–13.54 to –7.16)* |  |
|  | Increased sweating | *–3.67 (–5.07 to –2.27)* | *–5.22 (–6.61 to –3.83)* | *–8.08 (–10.77 to –5.38)* | *–4.02 (–6.77 to –1.28)* |  |  | *–5.58 (–10.77 to –0.4)* |  | *–3.79 (–5.85 to –1.72)* | *–6.25 (–9.36 to –3.13)* |  |
|  | Hot flashes | *–3.13 (–5.07 to –1.18)* | *–7.46 (–9.08 to –5.83)* | *–7.14 (–10.86 to –3.41)* | –3.72 (–7.66 to 0.21) |  |  | *–9.59 (–15.29 to –3.9)* |  |  | *–5.03 (–9.26 to –0.79)* |  |
|  | Nosebleeds | *–4.23 (–7.59 to –0.87)* |  | *–7.86 (–11.24 to –4.49)* |  |  | –5.73 (–15.03 to 3.57) |  |  | *–4.39 (–8.14 to –0.64)* | *–8.06 (–15.26 to –0.85)* |  |
|  | Body odor | *–7.77 (–13.31 to –2.22)* |  | –8 (–13.58 to –2.41) | 1.29 (–4.99 to 7.57) |  |  |  |  |  | –6.03 (–14.4 to 2.33) |  |

^a^Data adjusted for age, sex, ECOG, and treatment types.

^b^Italicized values are statistically significant (*P*<.05).
